# Desire for and barriers to obtaining effective contraception among women experiencing homelessness

**DOI:** 10.1186/s40834-020-00113-w

**Published:** 2020-08-17

**Authors:** Elizabeth Corey, Stephanie Frazin, Samantha Heywood, Sadia Haider

**Affiliations:** Department of Obstetrics and Gynecology, University of Illinois at Chicago, 820 South Wood Street, Chicago, IL 60612 USA

**Keywords:** Contraception, Long-acting reversible contraceptives, Unplanned pregnancy, Homeless

## Abstract

**Background:**

Homelessness is a significant and growing problem in the United States. Women experiencing homelessness face unique challenges as they have high rates of unintended pregnancy. They often face significant barriers to obtaining effective contraception. This study aimed to explore the contraceptive preferences, desire for, and barriers to obtaining effective contraception among women experiencing homelessness.

**Study design:**

In this cross-sectional study, 54 women experiencing homelessness in Chicago who were at risk for unintended pregnancy were surveyed.

**Results:**

While 94% of the women experiencing homelessness surveyed desired avoiding pregnancy, most were using the least effective contraceptive methods. Among participants, 80% had health insurance, 75% had a high school diploma, and 90% knew where to obtain birth control. Despite these factors, participants faced barriers to obtaining contraception. One significant barrier was lack of comprehensive contraceptive counseling as 62% report a physician had never counseled them on LARC methods. Approximately half of participants desired or would consider using a LARC in the future.

**Conclusions:**

Women experiencing homelessness in our study demonstrate significant unmet needs for effective contraception. Women experiencing homelessness would benefit from comprehensive contraceptive counseling and improved access to effective forms of contraception.

A homeless individual is defined as one who lacks a fixed, regular and adequate nighttime residence [1]. In the United States, homelessness remains a substantial and increasing problem. The odds of becoming homeless are 1:194, and over a half a million individuals are homeless each night [1]. Overall, women and children remain one of the fastest growing segments of the homeless population. Women comprise 37% of homeless individuals, while families comprise up to 50% of the homeless population. In the vast majority of homeless families, women lead the household as single parents [1].

Nearly 75% of pregnancies among women experiencing homelessness are unintended compared to 45% nationally [2]. A limited number of studies have explored risk of unintended pregnancy among women experiencing homelessness [3, 4]. Women experiencing homelessness have unique reproductive health needs given that having mistimed or unintended children can further perpetuate the cycle of poverty and homelessness. Approximately 13% of women experiencing homelessness are pregnant at any given time. This rate is more than double the rate of pregnancy among all American women and significantly higher than the 5% of housed low-income women who are currently pregnant [3]. In addition, women experiencing homelessness who become pregnant have an increased rate of complications.

Although previous research has shown that many women experiencing homelessness want to prevent pregnancy, they face significant barriers to obtaining effective contraception [4–6]. Previously cited barriers to contraceptives include fear of side effects, partner barriers, cost, storage, and lack of services on site [7]. Compared to the general population, women experiencing homelessness have decreased use of contraception and higher use of less effective contraceptive methods [2, 4, 6]. A survey of women experiencing homelessness in Los Angeles found that one-third of women experiencing homelessness used contraception rarely or never in the past year. Another study reported that 92% of women experiencing homelessness relied primarily on condoms and among those women only 32% reported condom use with every sexual encounter [4].

A potential solution to the increased rate of unintended pregnancies among women experiencing homelessness is ensuring access to comprehensive contraception counseling and to all methods of contraception including long-acting reversible contraceptives (LARC) if desired. Among women experiencing homelessness, other contraceptives may have higher failure rates than anticipated due to the daily challenges faced by women experiencing homelessness. There is little information on LARC use among women experiencing homelessness and LARC-specific barriers. One study explored the provider barriers to administering LARCs to women experiencing homelessness [8]. The survey suggested extremely limited access to LARCs among women experiencing homelessness. Only one-third of the organizations that provide healthcare services to women experiencing homelessness surveyed directly provided either intrauterine devices (IUDs) or the contraceptive implant to their patients. The significant barriers identified include lack of provider training, lack of resources for placement, costs, lack of insertion equipment, and concerns about complications [8]. A second study used mixed methodology to explore barriers to LARC use among homeless youth. Barriers identified included distrust of healthcare personnel who provide contraceptive counseling and misconceptions such as the inability to remove LARCs at the users’ discretion [9]. This study was a preliminary study, had a small sample size, and the results were not intended to be generalized. To our knowledge, no other study has focused on barriers to LARC use among women experiencing homelessness. Therefore, our study adds current knowledge to this topic as it assesses desire for and personal barriers to effective contraception, including LARCs, among women experiencing homelessness. Our hypothesis is that women experiencing homelessness have a strong desire for contraception, but are not regularly accessing reproductive health services.

## Methods

The Institutional Review Board (IRB) at the University of Illinois Chicago approved this study. After IRB approval was obtained, homeless shelters that serve women of reproductive age in Chicago were recruited to participate in our study through recruitment letters and phone calls. Researchers spent time developing long-term and trusting relationships with the local partners. The three Chicago-based homeless shelters that were chosen to participate in our study include New Moms, Sarah’s Circle, and The Night Ministry. The participants were recruited using a convenience sampling method and included both sheltered women and unsheltered women recruited through outreach services. Research personnel introduced our study at various programming events at New Moms. In addition, posters were placed throughout the shelter, and participants were asked to sign up at the front desk if interested. At Sarah’s Circle, research personnel and staff members were present during various hours at their day shelter in order to approach and recruit interested women. At The Night Ministry, participants were recruited at three of their homeless shelters that house women of reproductive age. Staff explained the study and asked women to sign up if they were interested. Research personnel also participated in their street outreach programs to recruit women currently living without shelter. The research was conducted from January 2016 to June 2017. In total, 54 women participated in the study.

To be eligible to participate, women had to be aged 18 to 45 years old and had to have spent at least one night in the past month in a homeless shelter, transitional shelter, public space, or vehicle. In addition, eligible participants were sexually active with at least one male partner in the last year. Participants were excluded if they were currently pregnant, unable to consent, if they had a tubal ligation or their primary partner had a vasectomy. The inclusion and exclusion criteria were designed to capture women experiencing homelessness who were currently at risk for unintended pregnancy. Among the participants who were identified and who met the inclusion and exclusion criteria, the response rate was 90%.

Participants were screened by research personnel in a private area of the shelter or mobile van in the case of the outreach programs in order to protect confidentiality. Care was taken to minimize coercion and undue influence by training research staff in informed consent, ethical considerations while working with vulnerable population, and study details. Therefore, research personnel provided written informed consent with the opportunity to ask questions for all participants.

After written consent was obtained, participants completed an online survey through REDCap on an iPad equipped with internet access. REDCap is a secure web application used to collect data. Although most women filled out the survey on their own, researchers were available to assist women by reading the questions and potential answers out loud if requested. It was suggested that these women move the iPad out of view from the researcher in order to continue to answer the question privately.

The survey contained approximately 60 question sets that focused on demographic information, homelessness, obstetric history, and experiences with contraceptives. The survey took approximately 30 min to complete. The survey was developed based on previous research questionnaires that were obtained [3, 6, 7]. Previously validated research questions were obtained from the National Survey of Family Growth and Pregnancy Risk Assessment and Monitoring [10, 11]. In addition, unique questions were created that were specific to women experiencing homelessness and LARC use, as standardized questions were not available. Participants were asked a series of closed-ended questions regarding desire for and barriers to both IUD use and contraceptive implant use. The surveys were translated into Spanish and a research personnel who was fluent in Spanish was available throughout the study. However, all participants in this study requested the English version.

Participants received a post-survey counseling session focused on either contraception education or preparing for a healthy pregnancy depending on their pregnancy desire. For women who desired avoiding pregnancy, this counseling session included a map with locations of Title X clinics in their neighborhood with details of which contraceptives were available at each location. All women received a $10 gift card to a local food store and two public transportation passes in order to provide transportation to visit the healthcare clinics if desired.

Statistical Package for the Social Sciences (SPSS) was used to calculate descriptive statistical analysis. Bivariate analysis was also performed utilizing the chi-square test and ANOVA. In regards to missed data, two individuals ended the survey early. The data associated with these participants was omitted from analysis. All other individuals completed the survey without missed data.

## Results

The participants’ demographic information is listed in Table 1. The mean age of participants was 28 years old. Among participants, 79% identified as black, 13% as white, and 4% as Hispanic. Approximately one quarter of participants were employed. Among the participants who were unemployed, the majority (73%) were looking for work. Eighty-three percent of participants described themselves as single without having been married. A high percentage of participants (68%) described themselves as chronically homeless, meaning they had been continuously homeless for one year or on 4 separate occasions in the past 3 years.

**Table 1:** Demographic Information of Participants

Demographic Variable	% Sample (*n* = 54)
*Ethnicity*	
Black	79
White	13
Hispanic	4
Asian	0
Other	0
*Education*	
None	2
1-4th grade	0
5-8th grade	0
Some high school	26
High school graduate	26
Some college	33
Technical, vocational	2
College degree	7
Masters or doctorate	4
*Marital Status*	
Married	2
Widowed	0
Divorced or annulled	11
Separated	4
Single, never been married	83
*Employment Status*	
Employed for wages	20
Self-employed	6
Out of work but looking for work	46
Out of work but not currently looking for work	17
Homemaker	2
Student	6
Military	0
Retired	0
*Health Insurance*	
None	20
State health insurance	74
Private health insurance	8

A large majority of participants desired to prevent pregnancy within the next year. When asked if they intended to avoid pregnancy in the next year regardless of housing status, 81% stated that they would like to avoid or would not mind avoiding pregnancy. If they remain homeless in the next year, 94% stated they would like to avoid pregnancy or would not mind avoiding pregnancy.

**Fig. 1:**
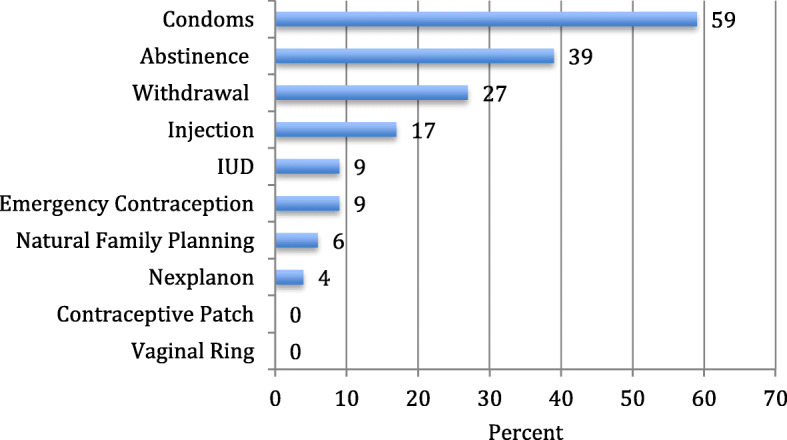
Current use contraception including all methods used among women experiencing homelessness in Chicago (n = 54)

**Fig. 2:**
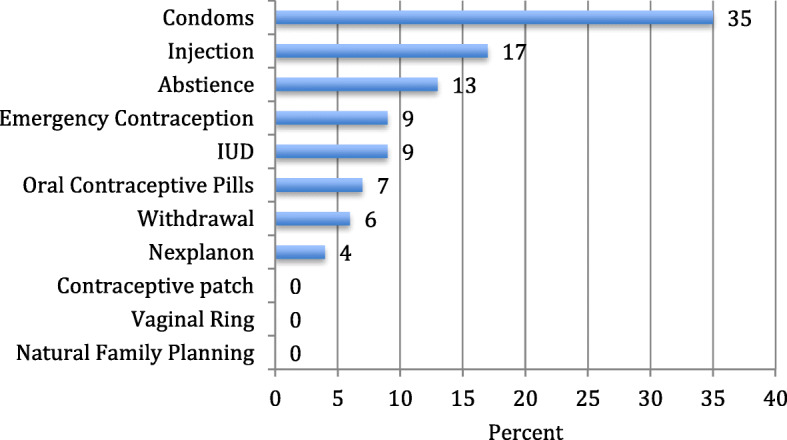
Current use contraception showing only the most effective method used among women experiencing homelessness in Chicago (*n* = 54)

Despite a desire to avoid pregnancy, the women experiencing homelessness surveyed were using the least effective methods in preventing pregnancy (Fig. 1). The most commonly reported methods selected when women were able to multi-select their current contraceptive use were condoms (58%), abstinence (38%), and withdrawal (27%). Figure 2 shows current contraceptive use filtering for the most effective method used for each woman. The most used method remains condoms with 35% of women using condoms as their most effective method of contraception (Fig. 2). Contraceptive methods were being used inconsistently with only 30% of participants stating that they had used contraceptives all the time in the past 6 months.

The participants had many assets that can be leveraged to assist them in obtaining contraception. Many participants had high levels of formal education with 72% completing high school or higher. Nearly 80% of participants had health insurance. The vast majority (90%) of participants knew where to go to access contraception and 83% were confident that they could consistently use birth control with every sexual encounter in the next 6 months.

**Fig. 3:**
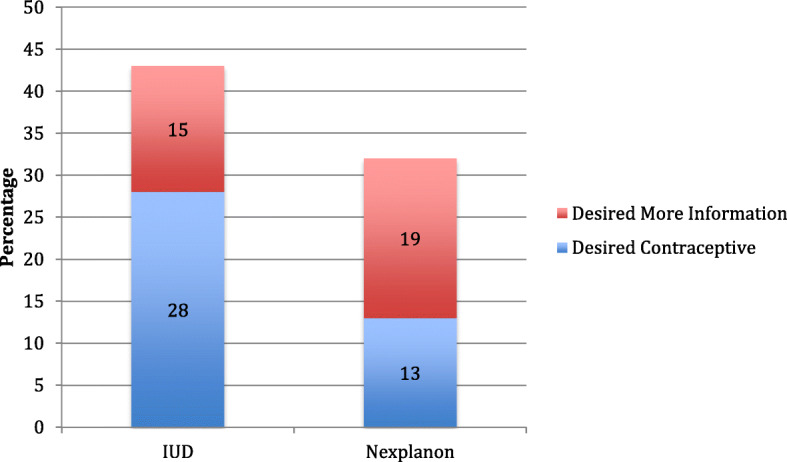
Desire for long-acting reversible contraceptives among women experiencing homelessness in Chicago

Participants were also asked about their desire for effective contraception, in particular their desire for LARCs. Approximately 43% of participants desired an IUD or desired more information about an IUD. One-third of participants desired a nexplanon or more information about a nexplanon (Fig. 3). In total, over half of participants desired a LARC or more information about LARCs. Women experiencing homelessness who had been pregnant previously were more likely to have heard of LARCs (*p* < .01) and women experiencing homelessness who had health insurance were significantly more likely to be using a LARC (*p* = .04). Age, race, education, and length of time women were homeless were not associated with current use or future desire for LARCs.

Participants expressed many concerns and barriers to obtaining effective contraception (Table 2). The most commonly cited barriers to contraception in general were concerns regarding side effects and the potential for birth control to negatively influence their health. In particular, among participants who did not desire LARCs, they cited a variety of reasons why they avoided LARC use (Table 3). The women were most commonly concerned about a foreign body being inserted into their body and complications with insertion.

**Table 2:** Most common concerns about birth control cited by women experiencing homelessness in Chicago

I am worried about the side effects of birth control	47%
I fear that birth control is harmful to my health	40%
My husband or partner does not like to use birth control, such as condoms	34%
Using birth control is uncomfortable	30%
I know what type of birth control to use	25%

**Table 3:** Most common concerns about LARC use cited by women experiencing homelessness in Chicago

I do not like the idea of something inside my body	81%
I worry there will be a complication with insertion	67%
I fear that the insertion of the device will be painful	62%
I worry about getting an infection from the device	57%
I worry about how these methods would change my bleeding pattern	43%
I fear that the device will cause infertility	40%
They are too expensive	27%

One important barrier to LARC use identified in this study is the lack of comprehensive contraceptive counseling by their healthcare provider. Seventy percent of participants reported that their main source of information about effective contraceptives is their healthcare provider. Most participants receive care at community health clinics (51%) or the hospital outpatient clinic (30%). However, the majority of participants (62%) report that they have not been counseled regarding LARCs by their healthcare provider.

## Discussion

The women experiencing homelessness in this study demonstrated a significant unmet need for comprehensive contraception counseling and access to contraception. Overall, women experiencing homelessness are at high-risk for unintended pregnancy. In our study, 94% of women experiencing homelessness surveyed would like to avoid pregnancy in the next year if they remain homeless. Yet, women in our study were more likely to be using the least effective contraceptive methods. This may help to explain previous research that found young women experiencing homelessness have a significantly higher pregnancy rate compared to young women in the general population [12]. Nearly 75% of pregnancies among women experiencing homelessness are unintended compared to 45% nationally [2, 4, 13].

We hypothesized that women experiencing homelessness did not have access to effective contraception because they were not regularly accessing reproductive healthcare services. However, our results indicate that women experiencing homelessness are seeking reproductive healthcare. The unexpected barrier we found is that the healthcare providers are not counseling women on the full spectrum of contraceptive options available.

Barriers exist both at the level of the provider and the healthcare organization. Saver et al. explored provider barriers to providing the full spectrum of contraceptive options to women experiencing homelessness. They surveyed 31 organizations in the national Health Care for the Homeless Practice-Based Research Network. Of the 17 organizations that provide contraceptives only 6 provided IUDs and 2 provided the contraceptive implant. Barriers cited include lack of provider training, resources and concerns regarding complications [8].

Where contraceptive services are available, healthcare providers need to employ comprehensive counseling for all their patients who would like to prevent pregnancy. A model involving shared decision making and patient-centered counseling should be employed. A chart or a graphic depicting all contraceptive can be utilized. For example, www.bedsider.org can be used as a resource to assist patients in finding and utilizing a method of birth control that best suits their needs.

If all contraceptive options are not available, healthcare providers should still counsel women on their options with referrals to other locations if desired. Providers should be aware that there is a tendency to counsel less effectively on methods that are unavailable at their location. Providers should counter this tendency by exploring all contraceptive methods with their patients and making referrals as necessary. Since the majority of women in our study received care at community health clinics, efforts should first be focused on these clinics to help ensure they offer the full spectrum of contraceptive options and counseling.

While counseling women experiencing homelessness, however, care should be taken to avoid coercion by the healthcare providers as women experiencing homelessness remain a vulnerable population. The counseling should therefore be patient-centered with the goal of enhancing informed choice through a shared-decision making framework. Future studies could be performed to assess the women’s perception of coercion and informed choice while receiving contraceptive counseling.

Many women in our study desired, but were not accessing long-acting reversible contraceptives. Participant-identified barriers should be addressed in comprehensive contraceptive counseling. Patients should be counseled on risks of insertion and side effect profiles. Providers should facilitate access and provision of LARCs when desired in an evidence-based fashion. For example, care should be taken to remove barriers, such as requiring two visits for a LARC insertion [14].

Furthermore, one quarter of participants cited money as a barrier to LARC use. As demonstrated in the Contraceptive CHOICE research project, when money was removed as a barrier, 75% of the women chose a LARC and the continuation rates were high [15]. Women experiencing homelessness who desire LARC use should be counseled on clinics that offer free LARCs through Title X funding or other avenues for subsidized contraceptive services.

The women experiencing homelessness in our study exhibited many assets that could be leveraged to assist the women in obtaining the full spectrum of reproductive healthcare. These include high levels of formal education, possession of health insurance, knowledge of where to access contraception, and confidence in ability to use birth control. As women experiencing homelessness are a heterogeneous group, the goals and assets of each individual should be evaluated and used to help empower her to meet her reproductive health goals.

Our study provides new information regarding limitations in contraception counseling and barriers to accessing effective contraceptive methods among women experiencing homelessness. However, some limitations should be considered. Our study is limited by a small sample size and a convenience sample that limits generalizability. Future studies are needed to further elucidate barriers that women experiencing homelessness face in accessing reproductive health care and barriers that healthcare personnel face in providing the full spectrum of contraceptive options. In addition, future studies should explore novel approaches aimed at increasing access to contraception for women experiencing homelessness with patient-centered approaches.

### Implications

Additional efforts by healthcare providers are needed to ensure women experiencing homelessness receive full contraceptive counseling while taking care to avoid coercion in this vulnerable population. Additional research should explore the most effective ways to deliver reproductive health services to women experiencing homelessness with subsequent programming initiated to apply the findings.

## Conclusion

This article adds to already existing literature as it demonstrated that the women experiencing homelessness in our study have a significant unmet need for effective forms of contraception. Approximately half of women experiencing homelessness surveyed desired a LARC or wanted additional information on LARCs. However, most women were not receiving comprehensive contraception counseling from their healthcare providers. Additional research and programming must be instituted in order to ensure that the reproductive needs of this vulnerable population are being met.

## Data Availability

The dataset used and analyzed for this study is available for review from the corresponding author upon reasonable request.
